# Resisting Oppression and Promoting Health Through Culturally Responsive Leisure Among Older Black Lesbians

**DOI:** 10.1093/geroni/igaf122.1070

**Published:** 2025-12-31

**Authors:** Austin Oswald, Lujira Cooper

**Affiliations:** Dalhousie University, Halifax, Nova Scotia, Canada; Board Member, Brooklyn, New York, United States

## Abstract

Numerous studies highlight the benefits of leisure participation for aging populations; however, the specific ways in which leisure contributes to the health and well-being of aging Black lesbians remain underexplored. Despite facing multiple forms of oppression at the intersections of age, race, gender, and sexuality, older Black lesbians actively engage in leisure as a means of self-preservation, community building, and resistance. This paper presents findings from a participatory action research project conducted with older Black lesbians to examine how leisure functions both as a site of oppression and as a tool for resistance. Guided by Black feminist theory, five Black lesbians between the ages of 65 and 75 participated in five focus groups, which were transcribed verbatim and analyzed using a reflexive thematic approach. Three themes were identified, highlighting the distinct leisure experiences of older Black lesbians and their need for culturally responsive leisure activities: (1) Reflecting realities: representation of diverse identities and experiences, (2) Belonging together: creating safe spaces for self-expression, and (3) Meeting needs: fostering culturally responsive leisure. These findings suggest that leisure is not only a source of enjoyment but also a critical mechanism for identity affirmation, social connection, and resilience. By exploring the intersections of identity, leisure, and health within the framework of structural oppression, this paper emphasizes the importance of adopting an intersectional perspective to reimagine leisure in ways that better serve marginalized and underserved populations. Implications for policy and practice interventions aimed at promoting healthy aging among older Black lesbians are discussed.

